# Impact of temperature on the bionomics and geographical range margins of the two-spotted field cricket *Gryllus bimaculatus* in the world: Implications for its mass farming

**DOI:** 10.1371/journal.pone.0300438

**Published:** 2024-04-30

**Authors:** Henlay J. O. Magara, Chrysantus M. Tanga, Brian L. Fisher, Abdelmutalab G. A. Azrag, Saliou Niassy, James P. Egonyu, Sylvain Hugel, Nana Roos, Monica A. Ayieko, Subramanian Sevgan, Sunday Ekesi

**Affiliations:** 1 International Centre of Insect Physiology and Ecology (*icipe*), Nairobi, Kenya; 2 Department of Feed Development, Madagascar Biodiversity Center Parc Botanique et Zoologique de Tsimbazaza, Antananarivo, Madagascar; 3 School of Agricultural Sciences and Food Security, Jaramogi Oginga Odinga University Science and Technology (JOOUST), Bondo, Kenya; 4 California Academy of Sciences, Entomology, San Francisco, California, United States of America; 5 Inter-African Phytosanitary Council of African Union (AU-IAPSC), Yaoundé, Cameroon; 6 Faculty of Science and Education, Busitema University, Tororo, Uganda; 7 Institut des Neurosciences Cellulaires et Intégratives, UPR 3212 CNRS, Université de Strasbourg, Strasbourg, France; 8 University of Copenhagen, Department of Nutrition, Exercise and Sports, Frederiksberg C, Denmark; Okayama University, JAPAN

## Abstract

*Gryllus bimaculatus* (Orthoptera: Gryllidae) is widely considered an excellent nutrient source for food and feed. Despite its economic importance, there is limited information on the impact of temperature on the bionomics of this cricket to guide its effective and sustainable mass production in its geographical range. The biological parameters of *G*. *bimaculatus* were investigated at eight different temperatures ranging from 20–40˚C. The Insect Life-Cycle Modelling (ILCYM) program was used to fit linear and non-linear functions to the data to describe the influence of temperature on life history parameters and its farmability under the current and projected climate for 2050. Our results revealed that *G*. *bimaculatus* was able to complete its lifecycle in the temperature range of 20°C to 37°C with a maximum finite rate of population increase (= 1.14) at 35°C. The developmental time of *G*. *bimaculatus* decreased with increasing temperature. The least developmental time and mortality were attained at 32°C. The highest wet length and mass of *G*. *bimaculatus* occurred at 32°C. The lowest temperature threshold for *G*. *bimaculatus* egg and nymph development was approximated using linear regression functions to be at 15.9°C and 16.2°C with a temperature constant of 108.7 and 555.6 degree days. The maximum fecundity (2301.98 eggs per female), net reproductive rate (988.42 daughters/ generation), and intrinsic rate of natural increase (0.134 days) were recorded at 32°C and the shortest doubling of 5.2 days was observed at 35°C. Based on our findings *G*. *bimaculatus* can be farmed in countries with temperatures ranging between 20 and 37°C around the globe. These findings will help the cricket farmers understand and project the cricket population dynamics around the world as influenced by temperature, and as such, will contribute to more efficient farming.

## 1. Introduction

The edible two-spotted black field cricket *Gryllus bimaculatus* De Geer is widely distributed globally [1–3]. This cricket is rich in protein, fat, minerals, and vitamins [4], which makes it an excellent ingredient for human food and livestock feed [5, 6]. Currently, *G*. *bimaculatus* is gathered from the wild when in season [7]. To mitigate food insecurity, and protein deficiency and generate income for both small and large-scale farmers, there are ventures around the globe to establish technologies to farm *G*. *bimaculatus* [8]. Prior investigations demonstrated that *G*. *bimaculatus* can be farmed at a temperature of 30°C. It is reported that the developmental period for *G*. *bimaculatus* egg through the nymph stage into the adult stage ranges between 37–45 days [9]. However, comprehensive knowledge of *G*. *bimaculatus* performance in different rearing temperatures is required. Moreover, its appropriate geographical range needs to be established to make an informed decision about farming and conserving it.

Different temperatures influence developmental time, adult longevity, development rate, growth rate, survival rate, sex ratio, fecundity, population growth, feeding and distribution of insects [10, 11]. Insect physiological processes depend on enzyme reactions that follow nonlinear curves in different temperatures [12] and reach a maximum point at an optimum temperature (T_opt_) [13]. Although all insects share this general trend, the actual shape of the temperature performance curve differs from one species to the other [14–16]. Moreover, this can be replicated in different life history parameters such as the rate of development and growth of a particular species of insect [17]. In edible crickets, temperatures associated with a faster rate of development produce adults of maximum size and weight [18, 19].

Insect Life Cycle Modelling package version 3.0 (ILCYM), is a free-of-charge online software package one can use to analyze insect population growth parameters data. The ILCYM helps to generate phenology models based on specific thermal-controlled experimental data by fixing functions for temperature-dependent bionomics of a particular insect species, which include development, developmental rate, mortality, fecundity, adult longevity, senescence, and life table parameters by compiling them into a general specific insect model. Previously ILCYM has been applied to predict the population growth parameters of many pest insects such as potato tuber moths, stemborers, armyworms, coffee pests, fruit flies, and pest parasitoids [17, 20, 21]. However, most recently, Otieno et al. [22] have used ILCYM and climaX data successfully to predict the population growth characteristics and map the distribution of a newly discovered edible cricket, *Scapsipedus icipe* in Africa.

To appreciate the impact of temperature on the egg hatch and nymph growth and development of *G*. *bimaculatus* in different temperature regimes, we employ the ILCYM models and climaX data to study the expected effects under current and projected climate change scenarios. *Gryllus bimaculatus* has three different life stages that mark its development: an egg stage, a juvenile stage, and an adult stage, so we decided to focus on the metamorphosis of *G*. *bimaculatus*. We generate eight different temperature scenarios and study their effect on the growth and development of the life stages of *G*. *bimaculatus*. We focus on egg and nymph stages as their sensitivity to temperature change might be further increased due to their increased metabolic rate [13], but we run our simulations until the adult stage is reached. We hypothesize that ILCYM model simulations combined with climaX data will help identify the main effects of temperature-induced changes in *G*. *bimaculatus* early-life-history traits when temperature changes are forced into the models. Further, the effect of fitting variables should differ between stages of development, which will have experienced different temperature exposure. Then ILCYM permits the combination of the models of the cohort and climaX data to the GIS environment resulting in three generic distribution indices including Establishment Index, Generation Index, and Activity Index to be visualized. This makes it possible for the life-table parameters of a specific insect under study to be predicted over time for a particular area.

Therefore, understanding the performance of insects at various temperatures is vital for establishing optimum temperatures. Optimum rearing temperatures would allow maximum development and growth rates of the insects while minimizing mortality. The temperature may also influence feed consumption, warming price, and discharge of wastes from the farming process [23]. In addition, by regulating the temperature in edible insect farmhouses, farmers will manage to manipulate the rate of development of their insects to achieve quick output from their production units.

The objective of our study was to assess the effect of various temperatures on the performance of *G*. *bimaculatus* from the egg through the nymph and adult stages. The bionomics of *G*. *bimaculatus* studied included developmental time, rate of development, adult longevity, mortality, body length and wet weight of adult crickets, reproductive and life history parameters. Despit*e G*. *bimaculatus* being a cosmopolitan cricket [24] with a short lifecycle and highly nutritious, its farming, however, has been reported only in a few countries around the world. For example, it has been reported being farmed in Kenya (Africa) [25], Indonesia [26], Thailand [27], Malaysia [28], Singapore [29] and South Korea [30] in Asia. Therefore, modelling and mapping the obtained data for *G*. *bimaculatus* will help in predicting the areas around the globe where the cricket can be farmed to help mitigate malnutrition and food insecurity under the current climate and 2050 climate scenario. This will lead to an influx of more farmers in various countries. Currently, commercial cricket farming is done under controlled conditions including temperature. Therefore, the results of this study can also be applied in both indoor and outdoor enclosures for cricket rearing where temperature models matching climate are needed.

## 2. Materials and methods

### 2.1. *Gryllus bimaculatus* population

The population of *G*. *bimaculatus* was started from 360 mature female and 120 mature male crickets captured from the field in Homabay County, Kenya in 2016. *Gryllus bimaculatus* eggs used in this experiment were obtained from a cricket farm at the International Centre of Insect Physiology and Ecology (*icipe*), Nairobi, Kenya. Nymphs and adult *G*. *bimaculatus* were reared in well-ventilated plastic cages measuring 600 mm long x 450 mm wide x 450 mm deep and maintained in a rearing room at 30˚C, relative humidity of 70 ± 5%, and 12 light: 12 dark periods [31]. Both stages of *G*. *bimaculatus* were fed on an artificial diet ad libitum. The artificial diet was developed by mixing 20% wheat bran + 20% maize bran + 20% soybean waste meal +20% pumpkin leaves meal + 20% fish waste meal. The *G*. *bimaculatus* were provisioned with water in shallow plastic dishes with a spongy material dipped in the dishes to prevent the crickets from drowning. The crickets obtained the water by sucking the spongy material. Further, moistened cotton fibre balls measuring 5 cm in diameter were introduced in the cages as the egg-laying medium [18]. The *G*. *bimaculatus* colony was replenished three times per year with an F1 generation of *G*. *bimaculatus* trapped from the grasslands in western Kenya to reduce inbreeding. The cricket colony was bred for over eight generations to build up a stable colony before the start of the experiments.

### 2.2. Effect of different temperatures on the developmental time of immature stages of *Gryllus bimaculatus* and adult longevity

Before the extraction of freshly laid eggs (less than 2 hours old), the moist cotton fibre balls containing the eggs were carefully removed from the cage into a holding container made of plastic and taken to the laboratory. Each ball of cotton fibre was then unwrapped and separated into thin cotton sheets for easy dislodging of the eggs using a camel hairbrush. The individual extracted egg was placed in a moist cotton spread-out in a rectangular plastic container measuring 160 mm long x 100 mm wide x 70 mm deep [31]. Each egg was then covered with a thin moistened sheet of cotton fibre followed by a reusable piece of cotton cloth to maintain a high level of relative humidity in the container. Every container was capped with a ventilated lid. The containers with the eggs were randomly distributed in incubators (SANYO MIR-553 and MIR-554, Sanyo Electrical Ltd., Tokyo, Japan) set at the following eight constant temperatures: 20, 25, 27, 30, 32, 35, 37, and 40°C, 70 ± 5% RH and 12 light: 12 dark periods [22]. These temperatures were selected on the basis that ILCYM has inbuilt models which can model even other temperatures not included which may impact bionomics of the cricket. The number of eggs used during the experiment at each temperature was 100. The incubated eggs were checked every six hours per day to determine when they hatched into nymphs [25]. The nymphs were then separated from the cotton using a fine camel hairbrush and retained in the same containers for experiments (160 mm long x 100 mm wide x 70 mm deep) containing an artificial diet. The unhatched eggs were monitored for one more month, after which they were declared as being dead. Newly emerged nymphs were housed individually to prevent cannibalism. Their diet was changed every three days to prevent the crickets from being poisoned by fungi that could have developed on the feed. The individual nymphs were monitored daily to establish their moulting to the next nymphal stage and any mortality.

### 2.3. Effect of different temperatures on the reproduction of *Gryllus bimaculatus*

The newly emerged adults of *G*. *bimaculatus* were retained in temperatures similar to those of immatures while evaluating the female fecundity and adult longevity for both sexes. An adult female and male were selected randomly and then placed together into a plastic container measuring 160 mm long x 100 mm wide x 70 mm to permit mating. The paired insects were offered a gram of artificial feed and water in shallow plastic dishes with a spongy material dipped in them. Additionally, the pair of crickets was provisioned with a ball of cotton fibre 5 cm in diameter for egg laying for the period of reproduction and vitality as reported by [22]. The diet was exchanged after three days of feeding. The survival of *G*. *bimaculatus* males and females was crosschecked daily to establish their adult longevity. Each experiment was replicated 24 times.

### 2.4. Modelling the life table parameters of *Gryllus bimaculatus*

Temperature thresholds for the development and survival of *G*. *bimaculatus* were approximated using functions depending on temperature. The functions explained the relation existing between temperature and population parameters of *G*. *bimaculatus*. In this case, ILCYM (Insect Life Cycle Modelling Package version 3.0) was employed [32]. This is a free online software package that has built-in modules that permit the insertion of non-linear functions to explain the correlation between insect population growth parameters and temperature. The non-linear models for our study include the Logan 1 model for the developmental rate of eggs and nymphs; polynomial models 1 and 4 for mortality of eggs and nymphs; Modified exponential of degree 3 for age-specific-fecundity and Hilbert and Logan 3 for both the senescence of males and females [32, 33]. This further includes a stochastic function for variability in development times within a population among individuals [34]. Development of such phenology models requires knowledge of lower and upper developmental thresholds as well as data on development for each life stage [34]. By application of the stochastic simulation function in ILCYM, the life history characteristics like gross reproductive rate (GRR), net reproductive rate (Ro), intrinsic rate of increase (rm), average generation time (T), and doubling time (Dt) for the *G*. *bimaculatus* population were approximated [32]. During the simulation of the life history characteristics of *G*. *bimaculatus*, only temperatures 20, 25, 27, 30, 32, 35, and 37˚C were considered. This is because the cricket was able to complete its lifecycle in these temperatures. Additionally, the listed temperatures were selected on the basis that ILCYM has inbuilt models which can predict the life history parameters of the cricket even for other temperatures not included above. At 40 ˚C the few eggs that hatched, the nymphs could not survive as they were desiccated. Hundred individuals of the cricket at the egg stage were used for simulation per temperature. There were seven replicates for each temperature.

### 2.5. Effect of different temperatures on the rate of development of immatures of *Gryllus bimaculatus*

*Gryllus bimaculatus* preadult stages rate of development was calculated as developmental rate = 1/median of developmental time [35] and then graphed against temperature. The linear portion of the developmental rate curve was modelled using linear regression to determine a correlation between the rate of development and temperatures [32]. The linear model applied is given as:

r(T)=a+bT (1)

$$\text{Low-temperature}\;\text{thresholds:}\;T_{\min}=-\frac{a}{b}$$

$$\text{Temperature}\;\text{constant:}\;k=\frac{1}{b}$$

where *r(T)* is the rate of development at temperature *T* (°C) while a and b are the intercept and gradient, respectively, of the regression formula while k is the temperature constant (degree-day).

Data were considered as best described by a nonlinear Logan 1 model [33] than with a linear model when AIC was smaller and R² was larger [32]. A nonlinear Logan 1 model was inserted into the rate of development, *r (T)*, and temperature, *T* to find out the correlation between temperature and rate of development by using the Marquardt algorithm function [36]. The Logan model 1 was applied to data from both the eggs and nymphal stages.

The Logan model 1 is given as:

$$r(T)=Y\cdot \left(e^{p.T}-e\left(p.T_{max}-\left(\frac{T_{max}-T}{v}\right)\right)\right)$$ (2)

where *r(T)* is the rate of development at temperatures *T*, *p* is the integer of Logan 1 model parameters, *e* and *v* are constant values, *T*_*max*_ is the upper-temperature limit, while *T*_*min*_ is the lower-temperature limit [33]. The *T*_*max*_ which is the temperature for the shortest time for development was approximated from the Logan model 1 for both the eggs and nymphs and the entire development from egg to adult stages. The optimum temperatures (*T*_*opt*_) for temperature for lowest mortality (= highest survival) were approximated from the Logan model 1 for both the eggs and nymphs.

### 2.6. Effect of different temperatures on the rate of mortality of immatures of *Gryllus bimaculatus*

Mortality refers to the decrease in the number of organisms of one or more species as a result of death and is applied when determining population characteristics like development rate and survivorship [37]. In ILCYM, mortality is used to describe the reduction in the number of immatures of an organism while senescence is used to explain the decline of the population of both the males and females of an organism with old age [32]. The mortality rate of *G*. *bimaculatus* eggs and nymphs was determined at each temperature and integrated into 45 non-linear models. A model was considered to be significantly better in describing the data if AIC decreased and R^2^ increased. Based on R^2^ and AIC, polynomial models of degrees 1 and 4 offered the appropriate plot for the mortality of the eggs and nymphs respectively. The formulae of the two polynomial models are as follows:

$$\text{m}(T)=exp\left(b_{1}+b_{2}\cdot x+b_{2}\cdot x^{2}\right)$$ (3)

$$m(T)=exp\left(b_{1}+b_{2}\cdot x+b_{3}\cdot {\left(x\right)}^{\wedge }0.5\right)$$ (4)

where *m (T)* refers to the mortality rate at temperatures *T* (°C) while constants *b*_*1*_, *b*_*2*_, and *b*_*3*_ are polynomial models of degrees 1 and 4 parameters. The polynomial models refer to the natural exponential.

### 2.7. Effect of different temperatures on the female fecundity and adult senescence of *Gryllus bimaculatus*

*Gryllus bimaculatus* age-specific fecundity was well described by a modified exponential model of degree 3. Curves of the cumulative number of eggs laid and normalized female age were determined as a ratio of age in days divided by survival time median. The following equation was used to calculate the age-specific fecundity of the cricket:

$$O(x)=1-e^{-aT^{b}}$$ (5)

where *O*(*x*) refers to the frequency of cumulative oviposition to normalized female age *x*, *T* is the temperature, while constants *a* and *b* are the modified exponential model parameters [32].

The average rate of oviposition for a single female was determined for each temperature regime as the sum of eggs oviposited by the particular number of females divided by the total number of females involved. The Wang 8 formula was inserted to explain the link between temperatures and oviposition by *G*. *bimaculatus* females:

$$f(T)=1-\frac{H}{\left(exp\left(1+exp\left(-\frac{x-tl}{Bl}\right)\right)\cdot \left(1+exp\left(-\frac{Th-x}{Bh}\right)\right)\right)}$$ (6)

where *f (T)* is the female fecundity at constant temperatures *T* (˚C) studied; *x* refers to the maximum female fecundity while *T*_*opt*_ is the temperature at which maximum fecundity occurs; and constants *Tl*, *Th*, *Bl*, *Bh*, and *H* are other Wang 8 model parameters [38]. The sex ratio was determined by counting adult males and females that emerged in each temperature and converted into percentages.

Senescence refers to the ageing factor of an organism and is applied to determine population characteristics like adult female and male longevity and fecundity by females [37]. In the ILCYM program, senescence is applied to adults of both sexes while mortality is applied to immatures [32]. *Gryllus bimaculatus* adult senescence was determined as the inverse of the median of adult longevity of both sexes at different temperature regimes plotted against respective temperatures under study. The Hilbert and Logan 3 models [32] offered the best curves to describe both male and female senescence. The Hilbert and Logan 3 models are expressed as follows:

$$s(T)=\Psi \left(\frac{{\left(T-T_{min}\right)}^{2}}{{\left(T-T_{min}\right)}^{2}+D}-exp-\frac{T_{max}-\left(T-T_{min}\right)}{Dt}\right)+\theta$$ (7)

Where *s (T)* refers to the senescence rate of males and females Of *G*. *bimaculatus* at various temperatures *T* under study while *Ψ*, *T*_*min*_, *T*_*max*_, *D*, *Dt*, and *θ* are parameters of Hilbert and Logan 3 model.

### 2.8. The effect of different temperatures on the body length and wet weight of *Gryllus bimaculatus* adult males and females

Ten individuals of each sex were randomly picked from newly emerged adults from the experimental setup. Each cricket was put into a transparent plastic container (160 mm long x 100 mm wide x 70 mm deep) with lid ventilation (140 mm long x 80 mm) fixed with a wire screen of 0.5mm. Water was given to each cricket using cotton wool dipped in Petri dishes. The newly emerged adult *G*. *bimaculatus* was provisioned with the diet for three days to mature sexually thus when the whole body of the cricket attained its true colour. Consequently, the wet weight of individual female and male *G*. *bimaculatus* was measured using a digital scale with a precision of 0.01 g (Vetek AB, S.No. 96034, Sweden). Measurements of body length for randomly selected individual *G*. *bimaculatu*s were achieved by placing each insect into a transparent glass cup with a diameter of 60 mm and a height of 140 mm. The cup was then placed on a well-calibrated white paper, for tracing and measuring the body length of each cricket. Each cricket was given time to stop moving before measuring and recording the length of the cricket [18].

### 2.9. Projection of appropriate places around the globe for *Gryllus bimaculatus* farming and its number of generations per year

To project the conducive places around the world for *G*. *bimaculatus* farming and the possible number of generations it can reproduce yearly in the current climate (2023), we utilized the minimum and maximum temperatures obtained from climaX data for each month from the WorldClim version 2.1 repository [39]. This temperature information can be downloaded free of charge at http://www.worldclim.org/. On the other hand, we used temperature data of the SRES-A1B scenario to predict the areas with suitable climates for farming *G*. *bimaculatus* in the year 2050 [40]. By combining the simulated data of population growth characteristics of *G*. *bimaculatus* under different temperatures studied and data for temperature obtained for the two climatic repositories, we determined and did a mapping of the establishment index (EI) (suitable farming areas) and generation index (GI) for *G*. *bimaculatus* around the world under current and projected climates.

During mapping, the EI picks out the regions in the world that are suitable for *G*. *bimaculatus* farming under distinct climates. The EI is usually determined based on the daily survival of immature stages of the cricket and varies between zero and one. When the value is one, it means that some individuals in each immature stage will survive the entire year. Alternatively, the days in which a specific immature stage would die are summed up and averaged by 365 days.

The function adopted in calculating the EI is given below:

$$EI=1-X_{k}$$ (8)

where *X*_*k*_ refers to the number of days per year whereby mortality of the egg-adult stage (*k*) is supposed to be 100% when divided by the 365 days [32].

The GI on the other hand estimates the average number of generations that *G*. *bimaculatus* can reproduce yearly in a particular region with a specific temperature. The generation index was determined by dividing the approximated total number of days for each generation by 365 days. The GI was calculated by adopting the following formula:

$$GI=\frac{\sum_{i=1}^{365}\frac{365}{T}}{365}$$ (9)

where 365 days in a year were taken into account and *T* is the approximated time in days for the generations of the cricket for each Julian day *i* (*i* = 1, 2, 3,…, 365) [32].

### 2.10. Statistical analyses and modelling

The influence of different temperatures on *G*. *bimaculatus* time of development of the immatures, adult male and female longevity, adult body length and wet weight of both sexes and fecundity of the females, were compared using a one-way analysis of variance. The means obtained for each parameter of *G*. *bimaculatus* were separated using the Student-Newman-Keul’s test with a level of significance set at 0.05. in R Statistical Software (Version 4.5.2) [41]. Modelling of life history traits that are influenced by different temperatures was performed using ILCYM, version 3.0 developed by Tonnang et al. [32]. This program has an equations builder that fits non-linear equations which describe correlations between different temperatures and the insect life history parameters. The equation builder then employs built-in statistical criteria to combine with the data on the life history parameters species of insect e.g. *G*. *bimaculatus*, to select the most suitable equation to describe the temperature-dependent processes. Consequently, the program assembles the life history processes into a phenological function for the population of the insect under evaluation [32]. The Akaike’s Information Criterion (AIC) [42] describes the most suitable approximated statistical equation while the coefficient of determination R^2^, which is the part of variation described by the equation, is statistics used for equation selection. When the AIC is low, and R^2^ is high, the equation is considered to be the best [42]. Always a female ratio of 0.5 is considered for all the experimental temperatures evaluated.

## 3. Results

### 3.1. The Effect of different temperatures on the time of development of immatures and adult longevity of *Gryllus bimaculatus*

The developmental time of eggs and nymph stages and male and female adult longevity of *G*. *bimaculatus* were highly influenced by the temperature (P < 0.001) (**Table 1**). Eggs managed to develop into nymphs at 20–37°C but failed to hatch at 40°C. The number of days taken by the eggs to hatch into nymphs was impacted by temperature (F = 22048, df = 6, 596, P < 0.001). The shortest time for eggs to hatch was 5.9 for a temperature of 32°C while the longest time was 34.1 days at 20°C (**Table 1**). Nymphs developed in 27.1 days at 32°C and 186.7 days and 20°C **(Table 1)**. The highest adult female longevity was 84.2 days at 20°C, compared to 78.0 days for adult male longevity (**Table 1**). The distribution frequency for developmental time for eggs, nymphs, and adult males was best described by logit models whereby R^2^ = 0.82–0.93 and the AIC = 384.1–2276.9 (**S1 Table in S1 File**). On the other hand, the distribution frequency of adult female longevity was best illustrated by a CLL distribution than a sigmoid model (Female: R^2^ = 0.85, AIC = 1946.4) (**S1 Table in S1 File**).

**Table 1:** The mean (±SE) of developmental time of the immature stages and adult longevity in days of *Gryllus bimaculatus* reared under different temperatures.

Temperature (°C)	Development time of immatures (days)		Adult longevity (days)	
	Egg	Nymph	Female	Male
20	34.13±0.01a	181.54±0.02a	84.22 ± 3.48a	78.00 ± 1.03a
25	14.32±0.01b	67.87 ±0.01b	81.02 ± 1.02a	70.83 ± 0.34b
27	10.47±0.01c	53.70 ±.0.04c	79.11 ± 1.45a	67.48 ± 0.46b
30	7.21±0.01d	37.20 ±0.03d	62.04 ± 1.12b	64.28 ± 2.64b
32	5.93±0.01e	27.12 ± 0.02e	58.20 ± 0.45b	54.43 ± 1.98c
35	6.01±0.01e	33.66 ± 0.02e	38.01 ± 0.60c	34. 44 ± 1.96d
37	6.04±0.01e	33.69 ±0.02e	33.00 ± 1.56c	30.10 ± 0.94d

Means in the same column followed by the same letters are not significantly different (Student-Newman-Keul’s test (P<0.005).

### 3.2. Effect of different temperatures on the rate of development of immatures of *Gryllus bimaculatus*

**Fig 1:**
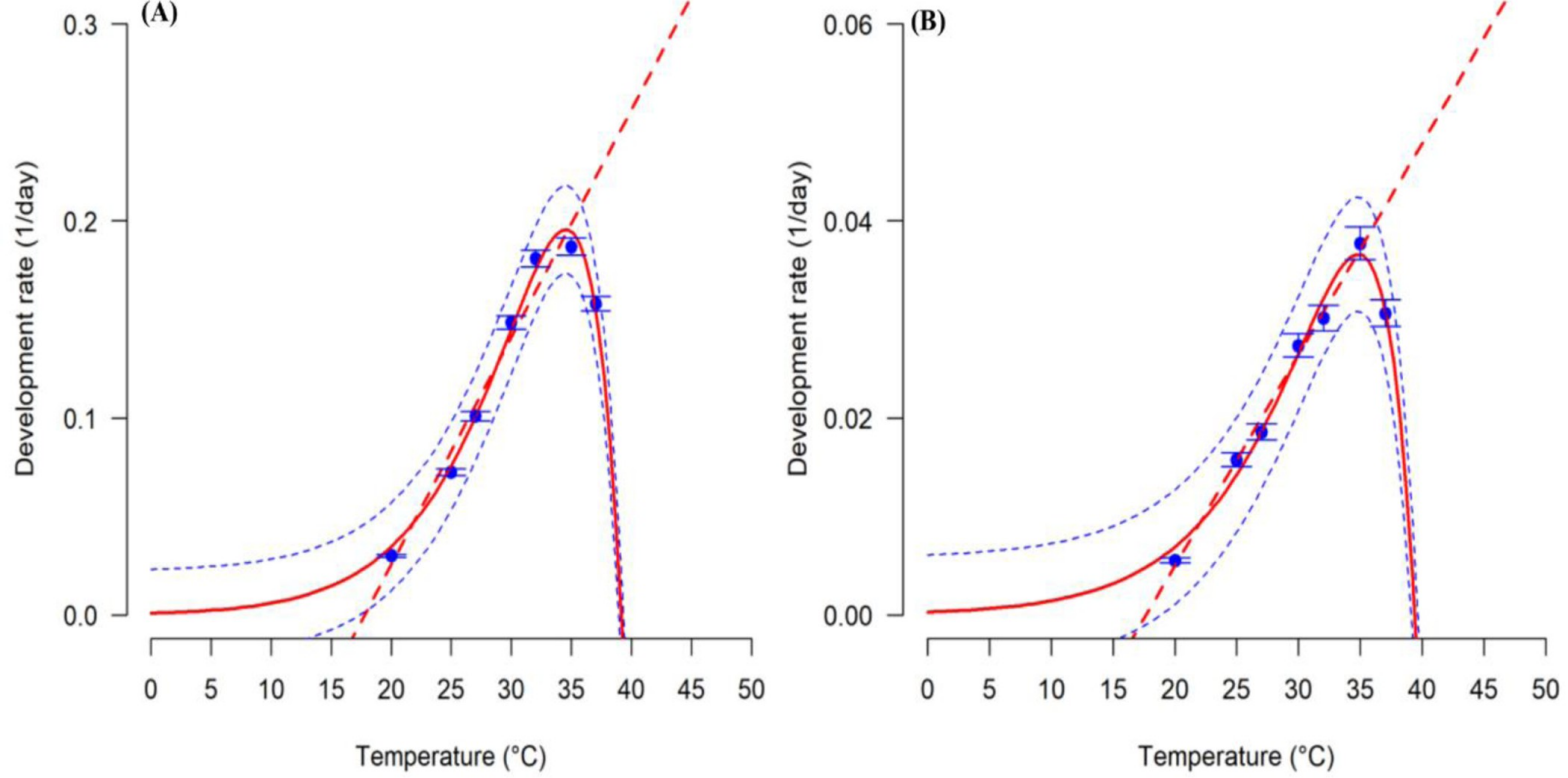
Relationship between temperature and developmental rate of *Gryllus bimaculatus* at the egg stage (A) and nymphal stage (B). The blue points are the observed values with bars representing the standard deviation. Fitted models are the dashed straight lines for linear models and solid lines for the Logan 1 model. Dashed lines in blue above and below represent the upper and lower 95% confidence interval.

Temperature effects on egg and nymphal stages of *G*. *bimaculatus* were estimated by use of the linear model to show the correlation between the rate of development and temperature (**Fig 1 and S2 Table in S1 File**). The lowest temperature thresholds for development (*T*_*min*_) were approximated at 15.9 for the egg and 15.5°C for the nymph stage (**Fig 1A and 1B and S2 Table in S1 File**). The thermal constants for development rate were 108.7- and 555.6-degree days for eggs and nymphs respectively. The best fit of the linear model was best described where R^2^ ranged from 0.86 to 0.90 and where the AIC ranged from -28.35 to -54.50 (**S2 Table in S1 File**). For too-low and too-high temperatures, the Logan 1 equation offered the most suitable fit for all immatures (R^2^ at 0.99 for both eggs and nymphs and where the AIC ranged between -45.52 and -64.35) (**Fig 1 and S2 Table in S1 File**). The highest temperature thresholds (*T*_*max*_) for the development of eggs and nymphs were approximated by the equation at 39.1 and 39.4°C, respectively (**Fig 1A and 1B**).

### 3.3. Effect of different temperatures on the mortality rate of immatures of *Gryllus bimaculatus*

**Fig 2:**
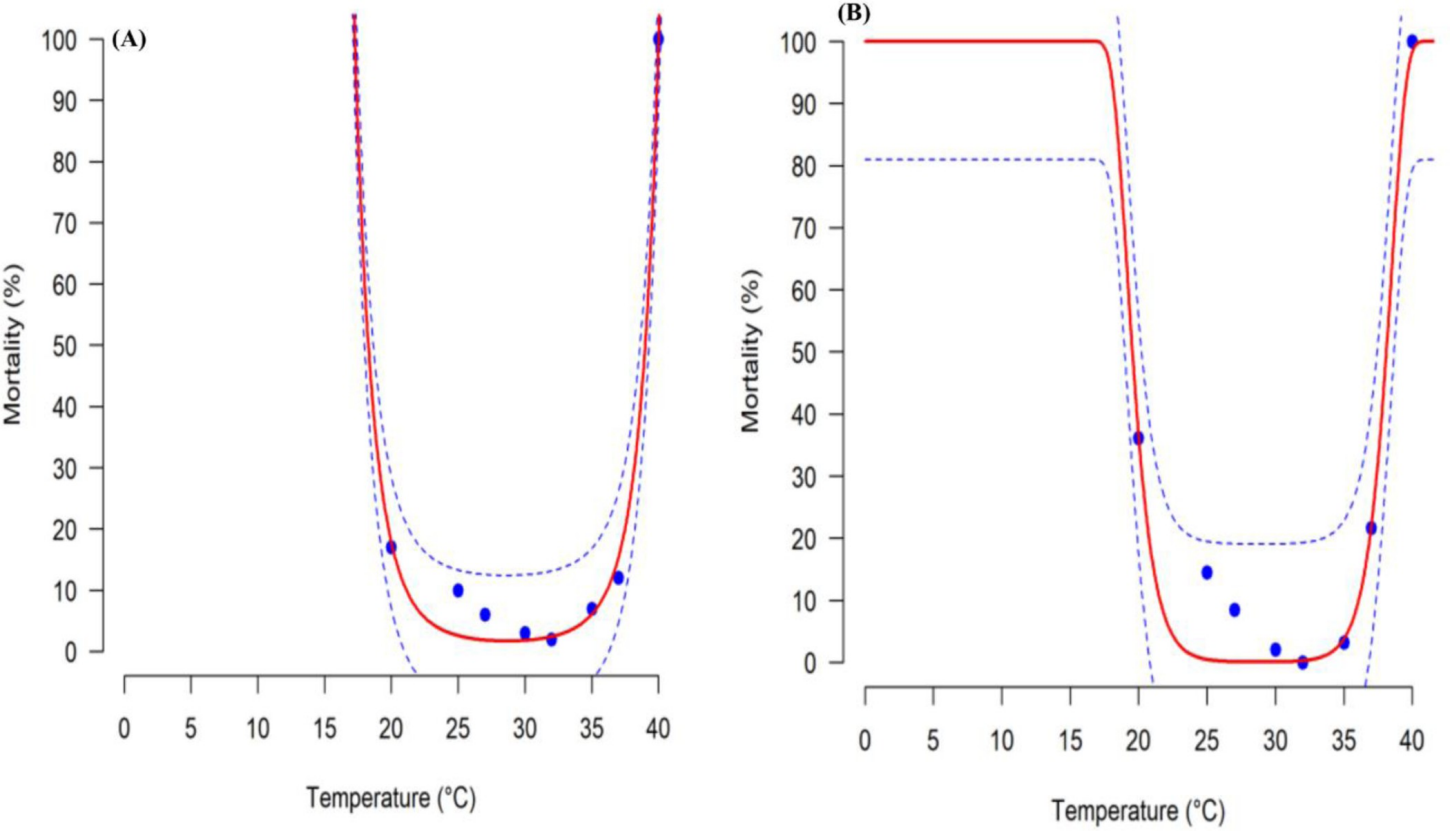
Relationship between temperature and the mortality rate of *Gryllus bimaculatus* egg (A) and nymph (B). Blue points are observed values. Solid red lines are fitted models, with a polynomial function of degree 4 for the egg, and a polynomial function of degree 12 for the nymph. Dashed lines in blue above and below represent the upper and lower 95% confidence interval.

Various temperatures influenced differently the rate of mortality of *G*. *bimaculatus* immatures (P < 0.001) (**Fig 2 and S3 Table in S1 File**). The temperature-dependent mortality of eggs was well illustrated by the polynomial equation of degree 1 where R^2^ = 0.99 and AIC = -22.13 **(Fig 2A and S3 Table in S1 File)**. A polynomial equation of degree 4 provided the best description for nymphal mortality where R^2^ = 0.87 and AIC = -4.77 (**Fig 2B and S3 Table in S1 File**). The optimal survival temperature for immatures of *G*. *bimaculatus* was estimated from the equation to be 32°C. A few eggs were able to hatch at 40°C however the hatched nymph did not survive in this temperature (**Fig 2A and 2B**). Despite the curve did not fit the data, the R^2^ of the models which measure the goodness of the fit was 0.99 and 0.87 for egg and nymphs respectively means that the accuracy of the fitting was 99% and 87%, which is acceptable. Thus, 99% and 87% of the variability within the data has been captured by the two models (**S3 Table in S1 File**).

### 3.4. Effect of different temperatures on oviposition periods, female fecundity, sex ratio and adult senescence of *Gryllus bimaculatus*

**Fig 3:**
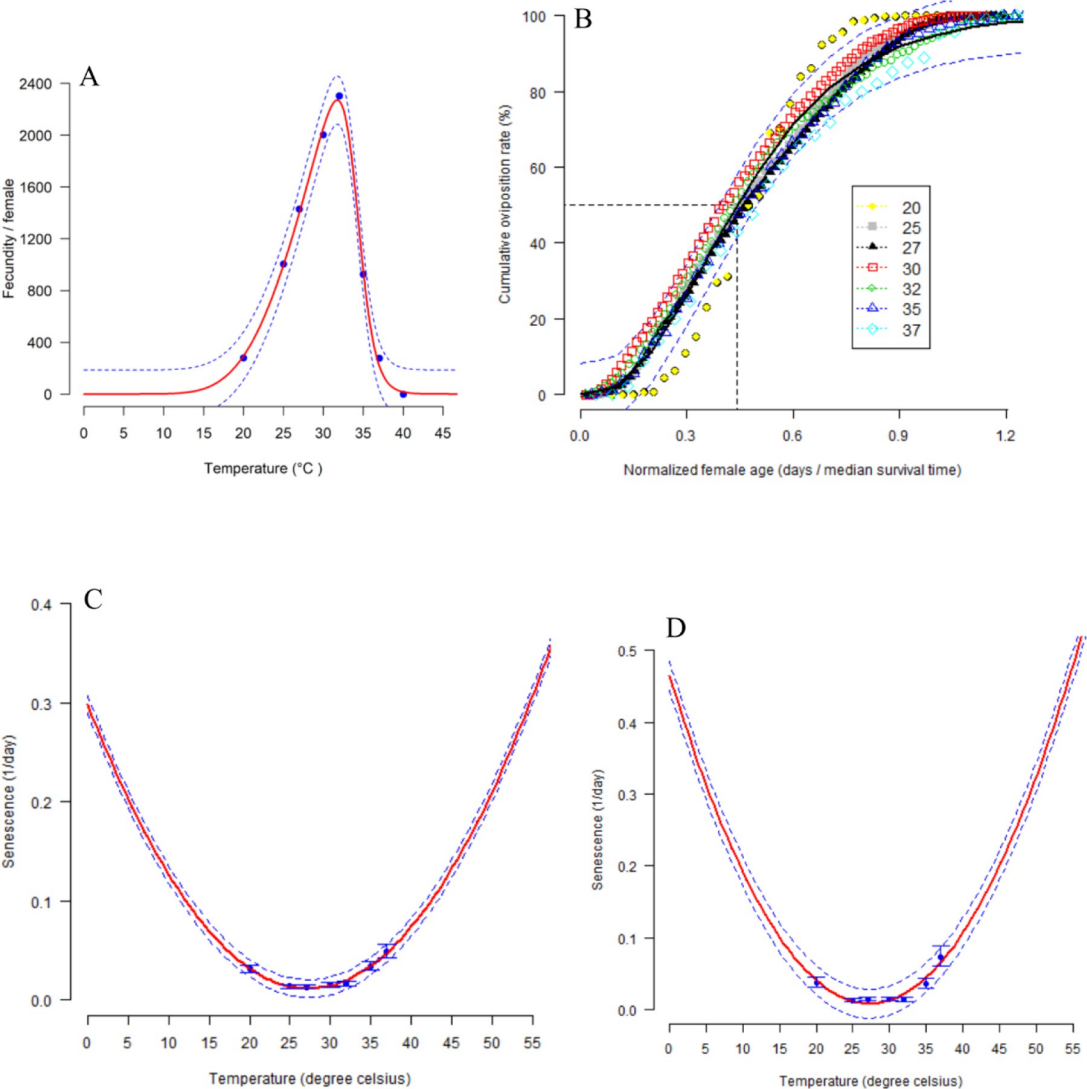
Relationship between temperature and fecundity fitted to polynomial function 12 model (A); temperature and cumulative oviposition rate fitted to exponential modified 1 model (B); temperature and adult female and male senescence rates fitted to Hilbert and Logan 3 model (C and D respectively) of *Gryllus bimaculatus*. Blue points are observed values with bars representing the standard deviation. Solid red lines are fitted models, with dashed lines in blue above and below representing the upper and lower 95% confidence interval.

Different temperatures studied had a significant impact on the preoviposition period (F = 114.2; df = 6, 284; P < 0.001), oviposition period (F = 775.9; df = 6, 284; P < 0.001), post-oviposition period (F = 143.5; df = 6, 284; P < 0.001) (**Table 2**) and fecundity (F = 4841; df = 6, 284; P < 0.001) in *G*. *bimaculatus* (**Fig 3A and S4 Table in S1 File**). The exponential modified function 3 provided the best description of the rate of age-specific cumulative oviposition where R^2^ = 0.99 and AIC = -1626.20. Female fecundity was minimum at 20°C, with 279.67 eggs per female, whilst fecundity was maximum at 32°C, where 2301.98 eggs were laid (R^2^ = 1.00 and AIC = 91.95) (**Fig 3B and S4 Table in S1 File**). The function showed that females of *G*. *bimaculatus* can deposit their eggs when subjected to temperatures 20, 25, 27, 30, 32, 35, and 37°C, with highest the female fecundity occurring at 32°C (**Fig 3B and S4 Table in S1 File**). Interestingly, the sex ratio of *G*. *bimaculatus* was male-biased at 20˚C, while that at 25, 27, 30, 32, 35 and 37˚C was female-biased (**S1 Fig in S1 File**). Both the male and female senescence of *G*. *bimaculatus* was greatly influenced by different temperatures (P < 0.001). Hilbert and Logan 3 function described the effect of temperature on the senescence of *G*. *bimaculatus* females and males (Female R^2^ = 0.93 and AIC = -37.51; Male R^2^ = 0.86 and AIC = -26.04) (**Fig 3C and 3D and S4 Table in S1 File**).

**Table 2:** The mean (days ±SE) of the preoviposition period, oviposition period, postoviposition, and fecundity of *Gryllus bimaculatus* reared at different temperatures under laboratory conditions.

Temperature (°C)	Parameter		
	Preoviposition	Oviposition period	Postoviposition
20	7.37 ± 0.29a	14.47 ± 0.14f	7.67 ± 0.36a
25	5.33 ± 0.23b	19.82 ± 0.39d	4.23 ± 0.21b
27	4.60 ± 0.19c	28.07 ± 0.32c	3.56 ± 0.12c
30	3.08 ± 0.11d	30.78 ± 0.33b	3.16 ± 0.11c
32	1.86 ± 0.14e	34.20 ± 0.29a	1.96 ± 0.07d
35	2.24 ± 0.17e	16.82 ± 0.40e	2.33 ± 0.14d
37	2.41 ± 0.11e	10.03 ± 0.19g	1.26 ± 0.08e

Means in the same column followed by the same letters are not significantly different (Student-Newman-Keul’s test: P<0.005).

### 3.5. *Gryllus bimaculatus* life table characteristics

Different temperatures had a significant impact on the simulated life history characteristics of *G*. *bimaculatus* (P < 0.001) (**Table 3 and S2 Fig in S1 File**). The r_m_ was highest at 32°C, with a value of 0.134 (**Table 3 and S2A Fig in S1 File**). Each adult female of *G*. *bimaculatus* had a GRR highest at 32°C, with 1073.7 daughters. Female crickets reared at 20°C and 37°C had the lowest GRR with 146.6 and 104.6 daughters per female respectively (**Table 3 and S2B Fig in S1 File**). The R_o_ varied between 67.2 and 988.4 daughters per female per generation at 20°C and 32°C respectively (**Table 3 and S2C Fig in S1 File**). The T (mean generation time) was longest at 20°C (185.2 days) and shortest at 35°C (44.6 days) (**Table 3 and S2D Fig in S1 File**). *Gryllus bimaculatus* population D_t_ was lowest (5.2 days) at 35°C, and highest (30.6 days) at 20°C (**Table 3 and S2E Fig in S1 File**). The highest λ was at 35°C, with a value of 1.14, and the lowest was at 20°C, with a value of 1.02 (**Table 3 and S2F Fig in S1 File**).

**Table 3:** Simulated life table parameters of *Gryllus bimaculatus* at various constant temperatures with the starting number of eggs (n) = 100). The intrinsic rate of increase (rm); Gross reproductive rate (GRR); Net reproductive rate (Ro); Mean generation time (T); Doubling time (Dt); and Finite rate of increase (λ).

Temperature (°C)	Life table parameters					
	r_m_	GRR	R_O_	T (days)	λ	Dt (days)
20	0.023 ± 0.001g	146.57 ± 18.91d	67.18 ± 12.04d	185.15 ± 1.67a	1.02 ± 0.001g	30.64 ± 1.46a
25	0.058 ± 0.004f	474.76 ± 48.82c	441.93 ± 48.06c	105.13 ± 6.49b	1.06 ± 0.004f	11.98 ± 0.77b
27	0.071 ± 0.001e	671.88 ± 29.60b	632.81 ± 34.04b	91.10 ± 0.74c	1.07 ± 0.001e	9.79 ± 0.15c
30	0.100 ± 0.001c	1009.49 ± 94.01a	930.03 ± 95.10a	68.09 ± 0.33d	1.11 ± 0.002c	6.91 ± 0.100de
32	0.134 ± 0.002a	1073.76 ± 101.65a	988.42 ± 84.25a	55.08 ± 0.31e	1.13 ± 0.002b	5.53 ± 0.06e
35	0.125 ± 0.001b	440.74 ± 37.92c	392.15 ± 32.37c	44.56 ± 0.29f	1.14 ± 0.003a	5.18 ± 0.08f
37	0.090 ± 0.008d	104.59 ± 11.22d	65.86 ± 12.60d	46.62 ± 2.40f	1.09 ± 0.009d	7.78 ± 0.72d
*F*	526.50	205.20	225.40	1575.00	520.10	807.20
*df*	6, 27	6, 27	6, 27	6, 27	6, 27	6, 27
*P*	0.001	0.001	0.001	0.001	0.001	0.001

Means in the same column followed by the same letters are not significantly different (Student-Newman-Keul’s test: P<0.005).

### 3.6. Effect of different temperatures on the body length and wet weight of adult males and females of *Gryllus bimaculatus*

**Fig 4:**
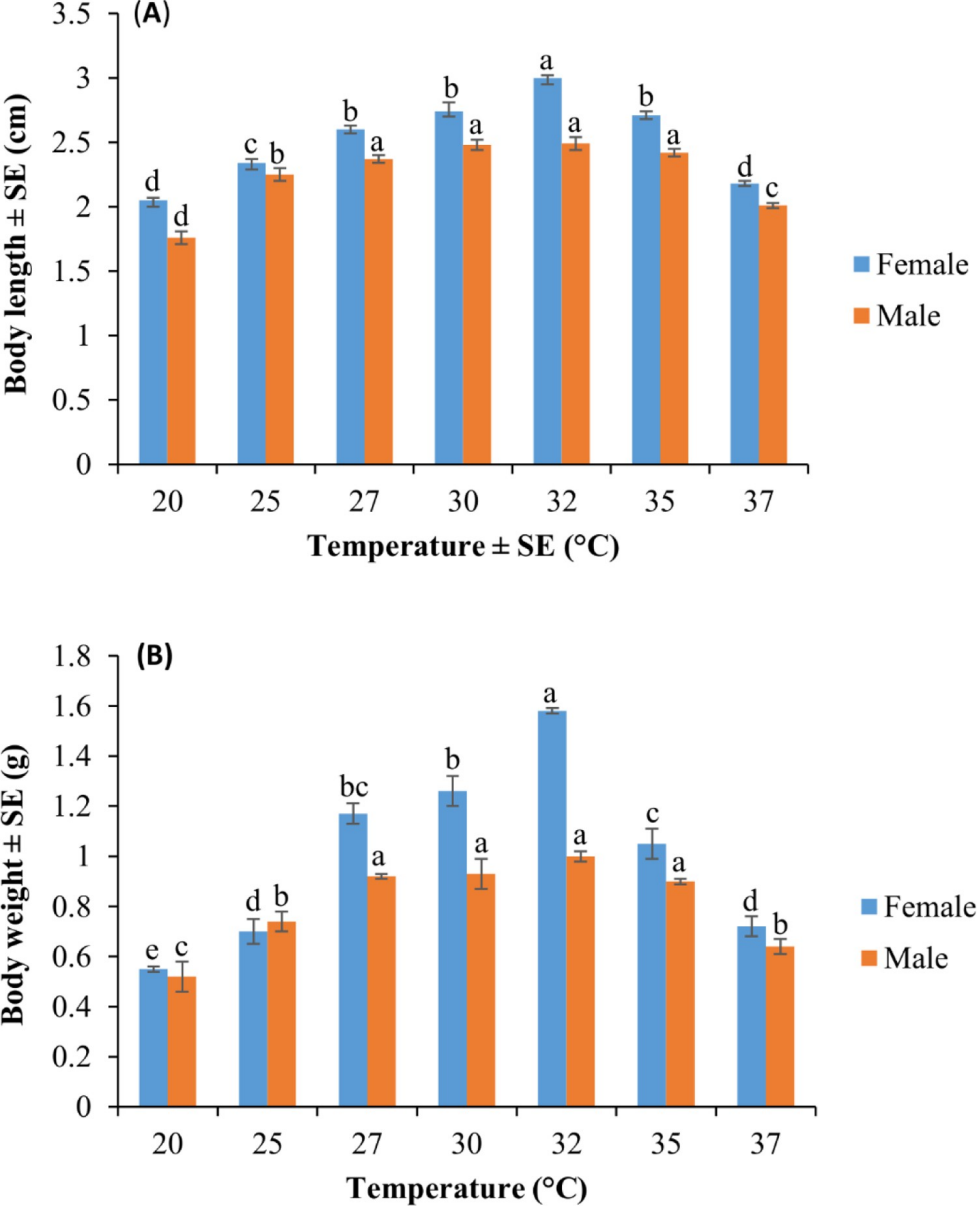
(A) Mean (±SE) body length and (B) Mean (±SE) wet weight of *Gryllus bimaculatus* females and males, respectively, at seven constant temperatures. Different letters indicate a significant difference while the same letters indicate no significant difference using Student-Newman-Keul’s test (P *<* 0.05).

There was an interaction among *G*. *bimaculatus* adult body length, sex of the cricket, and temperature (F = 4.70; df = 6, 126; P = 0.002). Different temperatures had a significantly high influence on male and female body length (male F = 53.36; df = 6, 63; P < 0.001; female F = 53.36; df = 6, 63; P< 0.001 (**Fig 4A**). The body length of female crickets was higher than that of male crickets at different temperatures (F = 127.07; df = 1, 126; P< 0.001) (**Fig 4A**). The *Gryllus bimaculatus* longest body length was noted at 32˚C for adult females and males while the shortest adult body length was observed at 20˚C (**Fig 4A**). In addition, there was a high interaction among wet body weight, cricket sex, and temperature (F = 13.13; df = 6, 126; P< 0.001). The body weight of *G*. *bimaculatus* males and females was significantly affected by temperature (male F = 21.03; df = 6, 63; female F = 69.63; df = 6, 63; P< 0.001) (**Fig 4B**). Further, female crickets had significantly heavier body weights than their male counterparts at various temperatures (F = 82.78; df = 1, 126; P< 0.001) (**Fig 4B**). The heaviest males and females were recorded at 32˚C (**Fig 4B**). There was a strong correlation between body length and wet body weight of *G*. *bimaculatus* (R = 0.96; P< 0.001).

### 3.7. Global potential farming areas of *Gryllus bimaculatus* under current and projected climatic conditions

**Fig 5:**
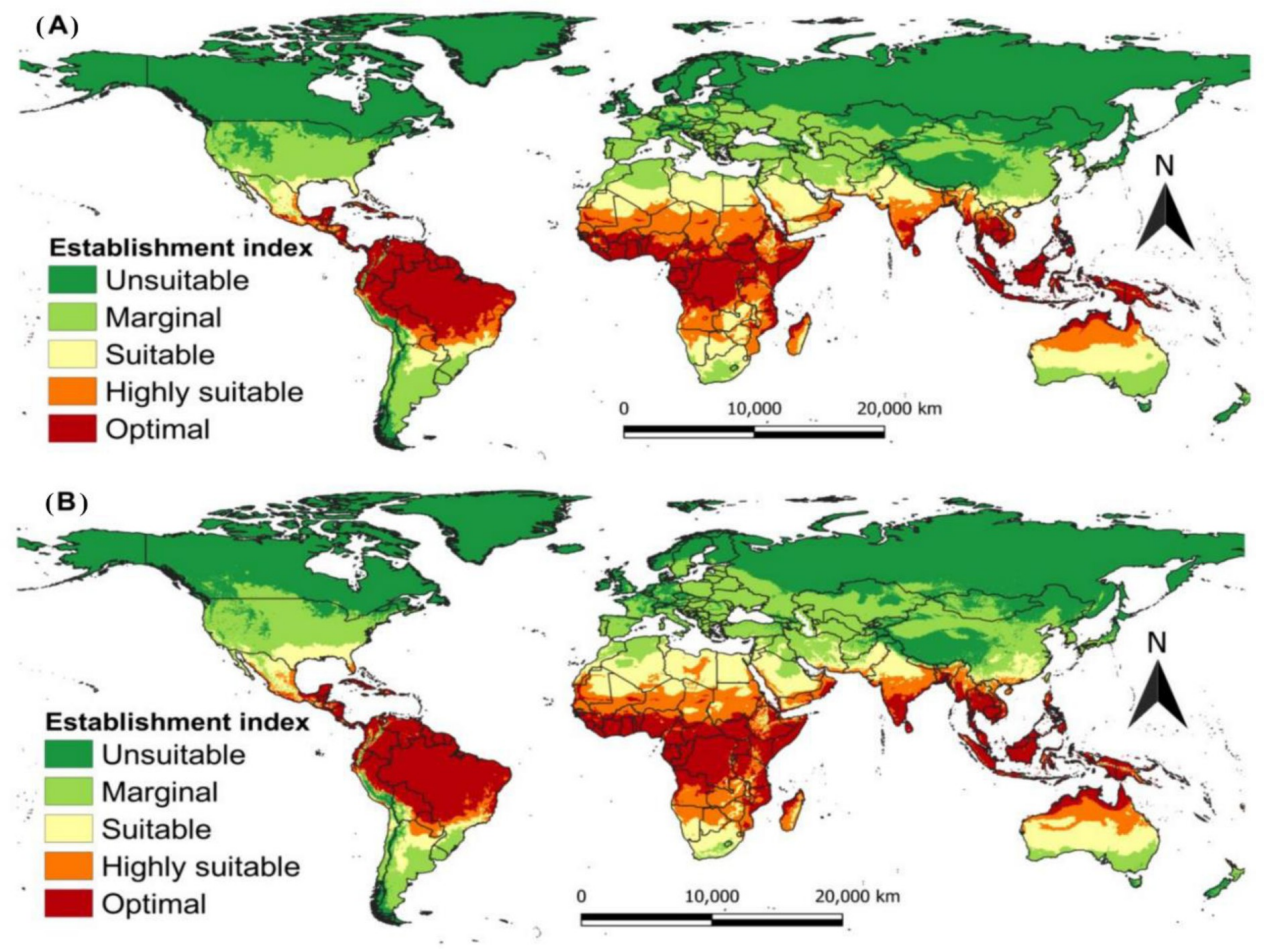
The Establishment Index (EI) for *Gryllus bimaculatus* modelled using CLIMEX in the A present time and CSIRO-Mk3.0 GCM running the SRES A2 scenario for 2050 B. The projections show a decrease in suitability around the equator and an increase in suitability in northern Europe and the Americas in 2050.

The potential farming areas of *G*. *bimaculatus* under current climatic scenarios aligned with the known distribution of the species around the globe. The projection function indicated vast regions around the globe where *G*. *bimaculatus* cricket can be farmed. The areas detected included the various countries in Africa, Asia, North, Central, and South America, Europe, and Oceania in the present year (**Fig 5A**).

In general, the climate changes projected for 2050 will expand highly suitable regions for *G*. *bimaculatus* farming (**Fig 5B**). Specifically, vast parts of South America, Sub-Saharan Africa, and a few places in Asia, Australia, and the southern portion of the USA are expected to become suitable for the two-spotted cricket in the future (**Fig 5B**). Therefore, the model has shown that less conducive places will shift to become moderately conducive areas for *G*. *bimaculatus* as one moves towards the poles and that highly conducive habits will increase near the equator.

### 3.8. Generations of *Gryllus bimaculatus* in the current and projected climatic conditions

**Fig 6:**
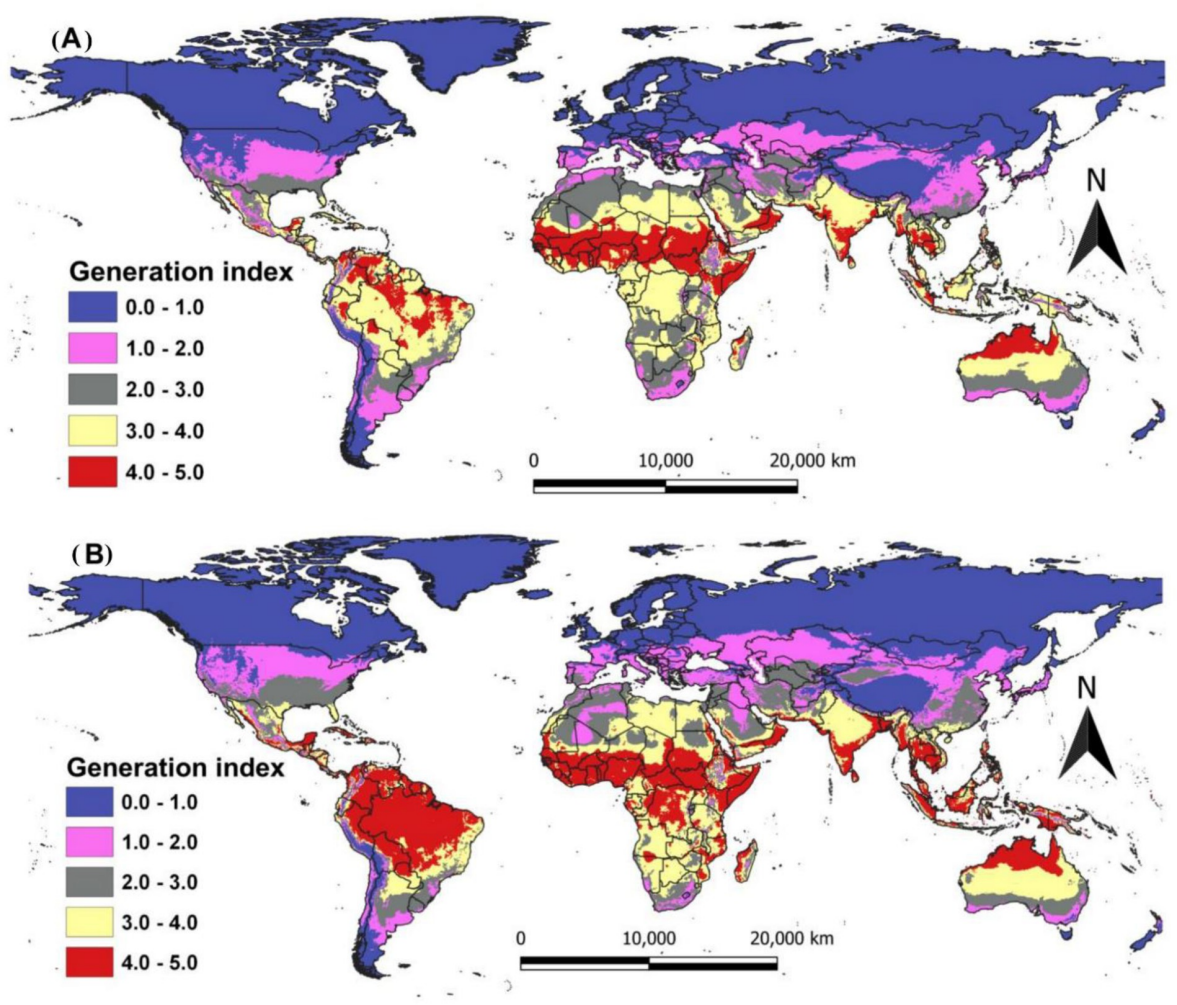
Current (A) and projected (B) generation index mapping of *Gryllus bimaculatus* farmability according to ILCYM model prediction in the globe.

The *G*. *bimaculatus* generation index shows the average number of generations that the cricket is likely to produce per year (**Fig 6**). Under current climatic conditions, the project demonstrates that *G*. *bimaculatus* could reproduce 3–5 generations yearly in countries within the tropics, with the number of generations decreasing towards the Sahara and Kalahari deserts in Africa and most parts of the USA, temperate, and Polar Regions (**Fig 6A**). Cricket could bear 3–5 generations per year in arid countries such as Libya, Tunisia, Morocco, South Africa, and many parts of the USA. In all European countries, cricket will give rise to one to two generations per year. Under future climatic conditions, an increase in the number of generations is expected in East, West, Central, Northern, and Southern African countries (**Fig 6B**). The same trend will be observed in Asia, South America, and the southern part of the USA, Australia, and Europe (**Fig 6B**).

## 4. Discussions

We report the effect of different temperatures on the bionomics of an edible cricket *G*. *bimaculatus* to guide its mass production globally. The current study showed that temperature had a remarkable influence on the development, growth, survival, and fecundity of *G*. *bimaculatus*. In addition, the study explains how climate change will affect suitable areas for cricket production under different temperature regimes.

The shortest developmental time of *G*. *bimaculatus* immatures was observed at 32˚C and the longest time for development was recorded at 20˚C. This could be because of increased metabolism at 32˚C as compared to at 20˚C. Complete development from egg to adult took between 1 and 7.2 months, in agreement with previous studies [8, 43] of the same species. In this study, the longer developmental time of immature stages of *G*. *bimaculatus* at the lower temperature (20°C) demonstrates that these temperatures are not ideal for adoption by farmers who want to rear the cricket for food and feed [19, 44]. By contrast, the short developmental period experienced at higher temperatures can hasten the maturation of the nymphs, which, in turn, would lead to an increased number of offspring and a corresponding increase in yield. The result is different from that of earlier workers who have reported an optimum temperature of 30˚C in *Acheta domesticus* [45] and *Scapsipedus icipe* [22]. As expected, the development of the crickets was greatly affected by temperature and showed temperature-dependent patterns. In terms of native distribution, *G*. *bimaculatus* is the second most widely distributed cricket across Europe, Africa, and Asia. It follows *A*. *domestica* which occurs in the United States of America, Europe, Oceania, and in a few African countries [1]. Other edible crickets such as *S*. *icipe* and *Modicogryllus conspersus* are restricted to Africa [19, 46].

The prime models explaining the correlation between time for development and change in different temperatures should be unimodal while approximating the low, optimum, and upper-temperature thresholds for *G*. *bimaculatus* [35]. The rate of development of *G*. *bimaculatus* peaked at 35°C, with *T*_min_ approximated at 16°C and *T*_max_ approximated at 39°C. These thresholds exhibit a typical temperature performance curve for all insects found in the tropics [22]. Although the optimum developmental rate was recorded at 35°C, the maximum size of the cricket was attained at 32°C. This finding is in line with the ecological rules relating to the temperature versus size rule, for many insect species. The rule predicts that the temperature that stimulated the maximum development rate of *G*. *bimaculatus* (35°C) did not promote a larger body size; a maximum body size was attained at 32°C [14]. At temperatures above optimal, the development rate of insects slows because of insufficient energy required for nymphal growth [10, 13]. At sub-optimal developmental temperatures, development rates in insects drastically decrease due to reductions in metabolic rates [12]. The Logan 1 function was the most suitable function to describe the correlation between the different temperatures and the rate of development of *G*. *bimaculatus* immatures. The findings of this study are in line with the report of Odhiambo et al. [47], who studied the influence of different temperatures on the developmental rate in *Acheta domesticus* cricket and confirmed that a change in temperature from 18˚C to 30˚C had a great influence on the time of nymph development, which could be reduced by 134.23 days in *A*. *domesticus*. This study further corroborates the finding of Otieno et al. [22] which indicated that increasing temperature from 20˚C to 37˚C decreased developmental time from eggs to adult females and males in *S*. *icipe* by 185.73 days and 190.29 days, respectively. The low-temperature thresholds in the linear equation agree with that of other insect species [21, 48]. At the optimum temperature thresholds, *G*. *bimaculatus* had a significantly high survival rate as well as a short generation time, key criteria for optimizing large-scale production [49]. For instance, Jerbi-Elayed et al. [50], showed that life history characteristics studied in constant temperatures gave vital data on the bionomics and ecosystem of insects, describing the changes that could occur within and between *G*. *bimaculatus* populations exposed to different temperatures.

Too low and too high temperatures are reported to exacerbate the mortality of insects while optimum temperatures lead to decreased mortality [10, 21]. In this study, mortality of both the eggs and nymphs was highest at 20˚C and it decreased as the temperature increased to 35˚C before it started to increase again. The lowest mortality was observed at 32˚C. The current study agrees with Odhiambo et al. [47] that tolerable temperatures for *G*. *bimaculatus* are directly proportional to the temperature change the cricket is subjected to. The high mortality observed at lower temperatures can be explained by inactivated enzyme reactions that are important in promoting the survival of *G*. *bimaculatus*, whereas at higher temperatures these enzymes are denatured, leading to death [13]. The polynomial model of degree 1 [32] explained well the mortality of the egg stage of *G*. *bimaculatus*, while the polynomial model of degree 4 [32] explained best the mortality of nymphal stages of *G*. *bimaculatus*. These models illustrated the temperature for the optimum survival of *G*. *bimaculatus* is 32˚C, which is higher than the optimal survival temperature for *S*. *icipe* at 30˚C [22], but lower than that of *Acheta domesticus* at 35˚C [47].

Temperature is reportedly to impact the sex ratio of crickets. In our study, the sex ratio was female-biased at higher temperatures (25, 27, 30, 35 and 37˚C) and strongly male-biased at lower temperature thresholds (20˚C). But at 32˚C the sex ratio was unbiased. Previously a similar influence of temperature on the sex ratio has been demonstrated in the cricket, *Scapsipedus icipe* [22], our findings give the first experimental report of temperature effects on the sex ratio of *G*. *bimaculatus*.

The oviposition period dropped with increasing temperature and stopped after 22 days at 32˚C for *G*. *bimaculatus*, compared to 9 days at 30˚C for *A*. *domesticus* [44]. The greatest number of eggs of *G*. *bimaculatus* were oviposited at 32˚C, for *A*. *domesticus* which is known to occur between 27–32 ˚C [9]. *Gryllus bimaculatus* egg-laying followed the pattern of *S*. *icipe* [22] and *Gryllus texensis* [51]. Our findings indicated that the *G*. *bimaculatus* female fecundity increased as body size increased, which is consistent with other insect species [48]. Previous studies have demonstrated that there is always a log-linear correlation between the size of the body and the number of eggs oviposited by cricket during the egg-laying timespan [52].

Temperature is known to markedly influence the body length and body weight of crickets [16]. Therefore, the determination of the optimum temperature to promote large body length and heavy weight in *G*. *bimaculatus* is an important factor for increased production, profits, and continuous farming of the cricket. The findings of this study have shown that *G*. *bimaculatus* female body length and wet body weight were higher than those of the males at different temperatures studied, but both female and male crickets experienced a decrease in these traits at 20, 25, and 37˚C, whilst the highest body length and body weight were observed at 32˚C. The increase in body weight and length recorded at 32˚C could be a result of an increase in feed consumption because at the optimum temperature metabolic rate is reported to be at its maximum point [51]. At each juvenile moult, cricket body length roughly doubles, leading to an exponential expansion of length from one juvenile stage to the other. This is because the body mass is found to accumulate in the last juvenile stage [53]. Whether the *G*. *bimaculatus* moulting is caused by particular factors, remains to be unraveled. Studies by [53] have, however, successfully explained that developmental pathways control body length by timed events in the *Manduca sexta* (L.) and *Drosophila melanogaster* Meigen. In *M*. *sexta*, a change of mass from about 1.2 grams to about 12 grams during its last juvenile stage contributed to about 90% of its final body mass [53]. Similarly, the last instar juvenile of *D*. *melanogaster* is reported to grow between 0.5 mg and about 1.8 mg, increasing by about 70% of its last mass [53].

The life history parameters of *G*. *bimaculatus* in the eight different experimental temperatures differed significantly. Total fecundity, the Ro and rm were highest at 32˚C. The shortest Dt was recorded at 35˚C and the time needed for the *G*. *bimaculatus* population to double at temperatures between 25 and 35˚C differed from 12.0 days to 5.2 days. The study further shows that temperatures below 25˚C will lead to increased Dt. The sites projected to be conducive by the function demonstrate that *G*. *bimaculatus* can endure various climates. This ability to endure different temperatures may elucidate to some extent the higher survival rate of nymphs. *Gryllus bimaculatus* reproduction remains one of the most crucial determiners of population vigour, particularly as *G*. *bimaculatus* reproduces most juveniles at an early age without giving them parental care. Appreciating the population growth factors of *G*. *bimaculatus* is important to help promote sustainable large-scale farming of the cricket for profits, provided these factors give the population growth rate of the cricket at current and projected generations [22]. The present work offers new scientific knowledge about life history characteristics and the biological potential of *G*. *bimaculatus*.

Our study represents the first experiment to model the potential farming areas for *G*. *bimaculatus* by employing its phenology data based on different temperatures under study. The functions show that *G*. *bimaculatus* can survive in different climatic conditions. The current and projected climate situation suggests that *G*. *bimaculatus* cricket can continue to be farmed in all countries where insects are eaten by local peoples as cultural heritage [54]. However, the projected changes in climate will impact *G*. *bimaculatus* negatively in some areas around the globe. Generally, the regions suitable for farming this cricket will expand by 2050, especially in countries close to the equator. For instance, some areas in South America, sub-Saharan Africa, and Asia will become conducive for farming the *G*. *bimaculatus* cricket. The USA and the northern part of Europe will become more conducive for farming *G*. *bimaculatus*.

Extrapolating to definite areas in the globe, our functions confirm the tropics are the most suitable areas for farming *G*. *bimaculatus*. From our findings, Africa is the leading hotspot followed by South America, Asia, Australia, and Oceania. The regions projected by the function were previously assessed for being hotspots where native people consume various insects as food or feed to livestock [1]. Although the farming areas in the globe for *G*. *bimaculatus* have not been fully documented, we hypothesize that these areas are similar to those reported in early studies by Magara et al. [52]. Therefore, this study provides a rationale to continue exploring the occurrence of *G*. *bimaculatus* in five of the world’s seven continents, to clarify the possibility of expanding *G*. *bimaculatus* farming to new regions. The environmental change seen under a projected scenario of the function indicated that *G*. *bimaculatus* can be farmed in warmer regions of the tropics and subtropics, where climate shifts would favour the population increase of successive generations of *G*. *bimaculatus* [40].

This study is the first report concerning *G*. *bimaculatus* population analysis and global mapping. Our findings will lead to a good understanding of the *G*. *bimaculatus* dynamics around the globe. However, we recognize the limitation of our projected model since the experimental insects were reared in different incubators. Future studies on modelling global climatic suitability for farming other reported edible crickets using temperature-dependent population growth and life history parameters should be conducted to fight malnutrition and promote food security.

## 5. Conclusions

The current work shows that *G*. *bimaculatus* can develop, grow, survive, and reproduce in various temperatures. The study also demonstrates that this cricket is suited to areas in the tropics and near the tropics however, the dynamics of the *G*. *bimaculatus* are likely to change by the year 2050 when it is likely to establish and farmed in new areas due to climate change. This study corroborates with early studies and offers a further description of rearing parameters that may be useful when farming *G*. *bimaculatus* in different parts of the world. These findings may be applied jointly with functions predicting the current and projected potential areas for farming cricket. Simulated bionomics for the cricket like mortality, adult female and male longevity, developmental rate, and female fecundity significantly influence the *G*. *bimaculatus* population structure, yield, and overall farming. As a result, these findings contribute to the body of knowledge needed to rear crickets at a large scale to mitigate global food insecurity and malnutrition. Our findings further reveal that *G*. *bimaculatus* is tolerant to different climates. Accompanying the idea that suitable areas for farming *G*. *bimaculatus* will increase by the year 2050, this cricket species forms a potential candidate for food production.

## Supporting information

S1 File (DOCX)

S1 Data set (XLSX)
