# Supplementary material for: Impact of temperature on the bionomics and geographical range margins of the two-spotted field cricket Gryllus bimaculatus in the world: Implications for its mass farming
Source: PLoS One. 2024 Apr 30;19(4):e0300438. doi: 10.1371/journal.pone.0300438 (PMC11060561; doi:10.1371/journal.pone.0300438)
Supplement: S1 File — (DOCX) [file pone.0300438.s001.docx]

Supporting information

**Impact of temperature on the bionomics and geographical range margins of the two-spotted field cricket *Gryllus bimaculatus*: implications for its mass farming**

**Henlay J. O. Magara ^1, 2, 3*^, Chrysantus M. Tanga^1^, Brian L. Fisher^2, 6^, Abdelmutalab G.A. Azrag^1^, Saliou Niassy^1^,** **James P. Egonyu^1^, Sylvain Hugel^2, 5^, Nana Roos^4^, Monica A. Ayieko^3^, Subramanian Sevgan^1^,** **and Sunday Ekesi^1^**

^1^International Centre of Insect Physiology and Ecology (*icipe*), Nairobi, Kenya; ^2^Department of Feed Development, Madagascar Biodiversity Center Parc Botanique et Zoologique de Tsimbazaza, Antananarivo, Madagascar; ^3^ School of Agricultural Sciences and Food Security, Jaramogi Oginga Odinga University Science and Technology (JOOUST), Bondo, Kenya; ^4^University of Copenhagen, Department of Nutrition, Exercise and Sports, Frederiksberg C, Denmark; ^5^Institut des Neurosciences Cellulaires et Intégratives, UPR 3212 CNRS, Université de Strasbourg, Strasbourg, France; ^6^California Academy of sciences, Entomology, 55 Music Course Drive, San Francisco, California, USA

*** Correspondence:** H.J.O. Magara, **Tel.:** + 254-721496376 Email: mhenlay@gmail. com

## Supporting Figures


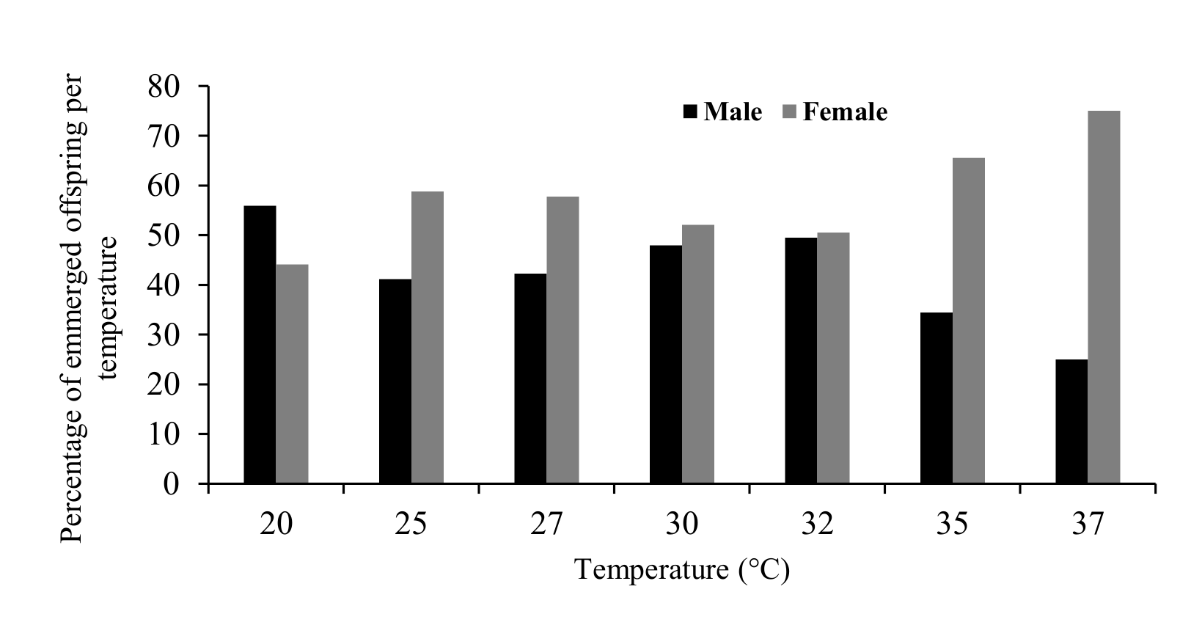


**S1 Fig.** Sex ratio of *Gryllus bimaculatus* adults that emerged from the last instar nymph reared at different constant temperatures.


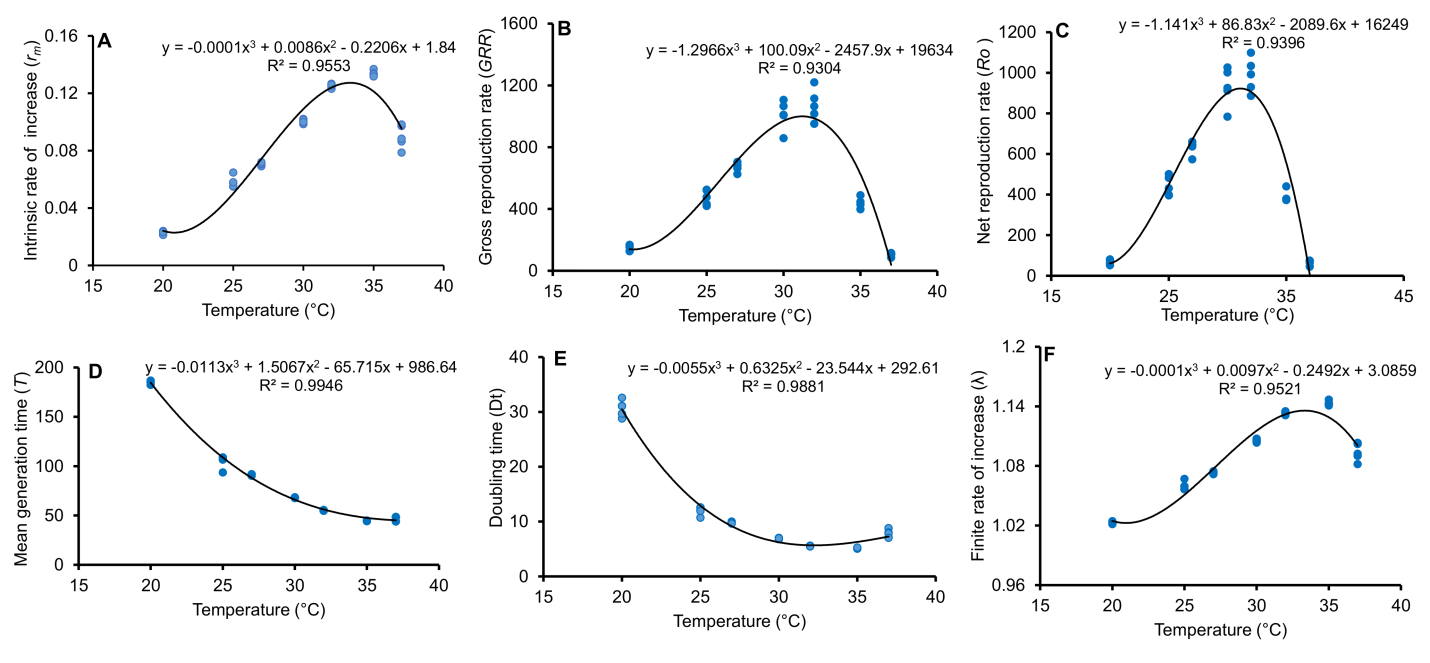


**S2 Fig.** Life table parameters of *Gryllus bimaculatus* through model prediction over seven different constant temperatures: Intrinsic rate of increase, rm (A); Gross reproductive rate, GRR (B); Net reproductive rate, Ro (C); Mean generation time, T (D); Doubling time, Dt (E); Finite rate of increase, λ (F).

## 2 Supporting Tables

**S1 Table.** Statistics of the goodness of fit and parameters estimated for logit and complementary clog-log (CLL) functions (*a* = y-intercept, *b* = common slope), fitted to cumulated frequency distributions for development time of immatures, and adult longevity for populations of *Gryllus bimaculatus* reared at different constant temperatures in the laboratory.

| Model parameters (mean± SE) | Temperature (°C) | Development of immatures  Egg Nymph | | Adult longevity  Female Male | |
| --- | --- | --- | --- | --- | --- |
| Intercept (a) | 20 | -111.11 ± 4.77 | -184.44 ± 4.83 | -17.86 ± 0.38 | -12.57 ± 0.31 |
|  | 25 | -83.18 ± 3.56 | -146.54 ± 3.85 | -22.04 ± 0.46 | -16.49 ± 0.39 |
|  | 27 | -72.76 ± 3.12 | -140.40 ± 3.68 | -22.15 ± 0.47 | -16.16 ± 0.39 |
|  | 30 | -60.40 ± 2.62 | -127.02 ± 3.34 | -22.58 ± 0.45 | -16.09 ± 0.38 |
|  | 32 | -53.26 ± 2.57 | -115.53 ± 3.03 | -21.11 ± 0.44 | -16.22 ± 0.38 |
|  | 35 | -54.22 ± 2.36 | -123.81 ± 3.25 | -17.40 ± 0.37 | -12.71 ± 0.31 |
|  | 37 | -58.49 ± 2.48 | -123.17 ± 3.23 | -15.63 ± 0.34 | -10.12 ± 0.25 |
| Slope (b) |  | 31.71 ± 1.35 | 35.87 ± 0.94 | 5.04 ± 0.11 | 3.73 ± 0.09 |
| R^2^ |  | 0.93 | 0.90 | 0.85 | 0.82 |
| AIC |  | 384.13 | 921.84 | 1946.37 | 2276.85 |

**S2 Table.** Estimated parameters of linear and Logan 1 model for the effect of temperature on developmental rate (1/day) for egg and nymphal stages of *Gryllus bimaculatus.* R^2^ *(*"R squared") - Coefficient of determination; $T_{\min}$ - Lower limit temperature; K - Degree-day (DD) requirements; Y, p and v: Constant values; “P” refers to the number of model parameters while “n” and “m” are constant values; $T_{\max}$ - The maximum lethal temperature; $T_{\mathrm{opt}}$ - Optimum temperature for survival; “a” and “b” are estimated values of the intercept and slope.

| Stage | Model name | Model parameters (±SE) | | Statistics | | | | |
| --- | --- | --- | --- | --- | --- | --- | --- | --- |
|  |  |  |  | *F* *df p* R^2^  AIC | | | | |
| Egg | Logan 1 | Y  $T_{\max}$  $T_{\mathrm{opt}}$  p  v | 0.002 ± 0.001  39.132 ± 0.008  35.000  0.206 ± 2.006  4.365 ± 0.023 | 141.32 | 3, 6 | 0.001 | 0.993 | -45.52 |
| Nymph |  | Y  $T_{\max}$  $T_{\mathrm{opt}}$  p  v | 0.001 ± 0.041  39.379 ± 0.001  35.000  0.162 ± 0.011  3.471 ± 0.001 | 70.70 | 3, 6 | 0.003 | 0.986 | -64.35 |

**S3 Table**. Statistics of the goodness of fit and parameters of models fitted to the relationship between the mortality rate of *Gryllus bimaculatus* immatures and temperature. *F*: F-test statistic, *df*: degree of freedom, *p*: probability value, R^2^: coefficient of determination, and AIC: Akaike’s Information Criterion.

| Stage | Model name | Model parameters (±SE) | | Statistics | | | | |
| --- | --- | --- | --- | --- | --- | --- | --- | --- |
|  |  |  |  | *F* *df p* R^2^  AIC | | | | |
| Egg | Polynomial model 1 | b_1_  b_2_  b_3_ | 25.520 ± 0.108  2.121 ± 0.035  0.037 ± 0.001 | 176.78 | 2, 5 | <0.001 | 0.99 | -22.13 |
| Nymph | Polynomial model 4 | b_1_  b_2_  b_3_ | 91.271 ± 0.108  3.361 ± 0.048  -35.692 ± 0.281 | 16.83 | 2, 5 | 0.006 | 0.87 | -4.77 |

**S4 Table.** Statistics of the goodness of fit and parameters of models fitted to describe the relationship between *Gryllus bimaculatus* cumulative oviposition, mean total oviposition, and adult senescence and temperature. *F*: F-test statistic, *df*: degree of freedom, *p*: probability value, R^2^: coefficient of determination, and AIC: Akaike’s Information Criterion.

| Demographic parameters | Model name | Model parameters (±SE) | | Statistics | | | | |
| --- | --- | --- | --- | --- | --- | --- | --- | --- |
|  |  |  |  | *F* *df p* R^2^  AIC | | | | |
| Relative oviposition | Exponential modified 3 | a  b | 3.420 ± 0.055  3.229 ± 0.022 | 36437.65 | 1, 444 | <0.001 | 0.99 | -1626.20 |
| Average total oviposition | Wang 8 | T1  Th  B1  Bh  H | 31.748 ± 0.348  34.032 ± 0.167  10.229 ± 0.641  1.063 ± 0.102  -118719.520 ± 0.001 | 359.61 | 4, 7 | <0.001 | 1.00 | 91.95 |
| Male senescence | Hilbert and Logan 3 | *Ψ*  $T_{min}$  $T_{max}$  D  Dt  *θ* | 1908037.000 ± 0.001  27.296 ± 0.001  38.567 ± 0.001  310029011.000± 0.001  0.017 ± 0.001  0.008 ± 0.002 | 18.80 | 5, 15 | <0.001 | 0.86 | -26.04 |
| Female senescence |  | *Ψ*  $T_{min}$  $T_{max}$  D  Dt  *θ* | 2384449.000 ± 0.001  27.297 ± 0.001  38.567 ± 0.001  6190319659.000 ± 0.001  0.017 ± 0.001  0.011 ± 0.001 | 38.12 | 5, 15 | <0.001 | 0.93 | -37.51 |
